# Supplementary material for: Lipid Process Markers of Durum Wheat Debranning Fractions
Source: Foods. 2023 Aug 12;12(16):3036. doi: 10.3390/foods12163036 (PMC10453066; doi:10.3390/foods12163036)

Lipid Process Marker of Durum Wheat Debranning Fractions

Silvia Marzocchi [1,*], Maria Cristina Messia [2], Emanuele Marconi [3], Maria Fiorenza Caboni [1,4], Federica Pasini [1,4]

Figure S1. Chromatogram of the tocochromanols of I-WG sample. Peaks: (1) α-tocopherol; (2) α-tocotrienol; (3) β-tocopherol; (4) γ-tocopherol; (5) β-tocotrienol.

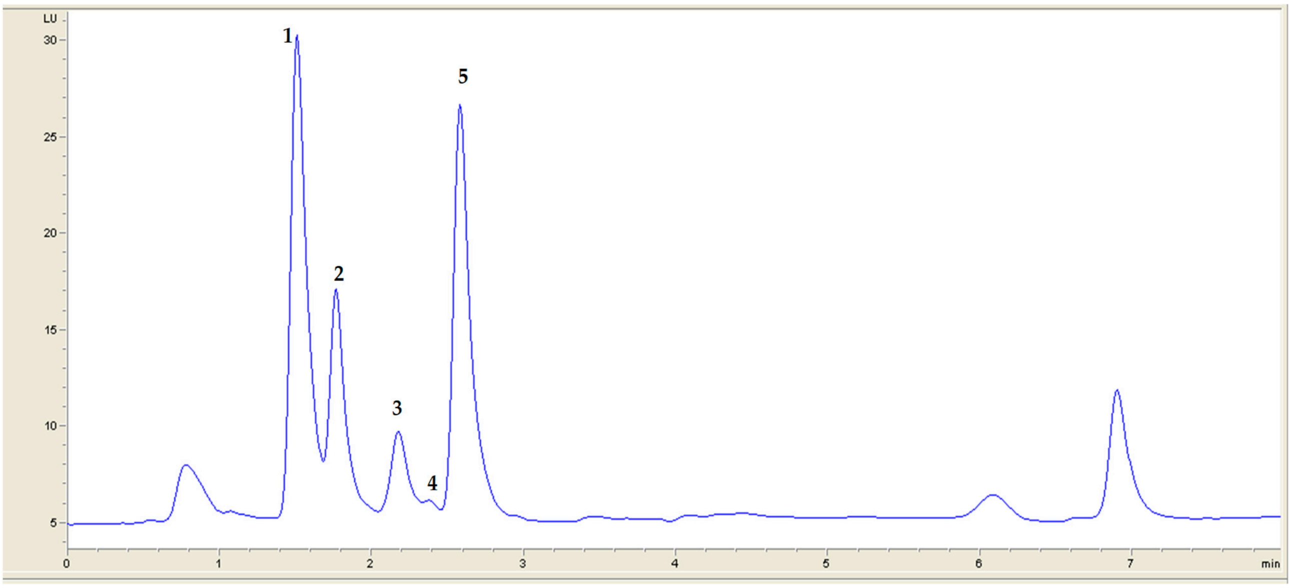

Figure S2. Chromatogram of the sterols of C-WG sample. Peaks: IS: 5α-cholestan-3β-ol (Internal Standard); (1) Campesterol; (2) Campestanol; (3) Stigmasterol; (4) β-sitosterol; (5) Sitostanol; (6) Δ^5-Avenasterol; (7) Avenastanol; (8) Δ^7-avenasterol.

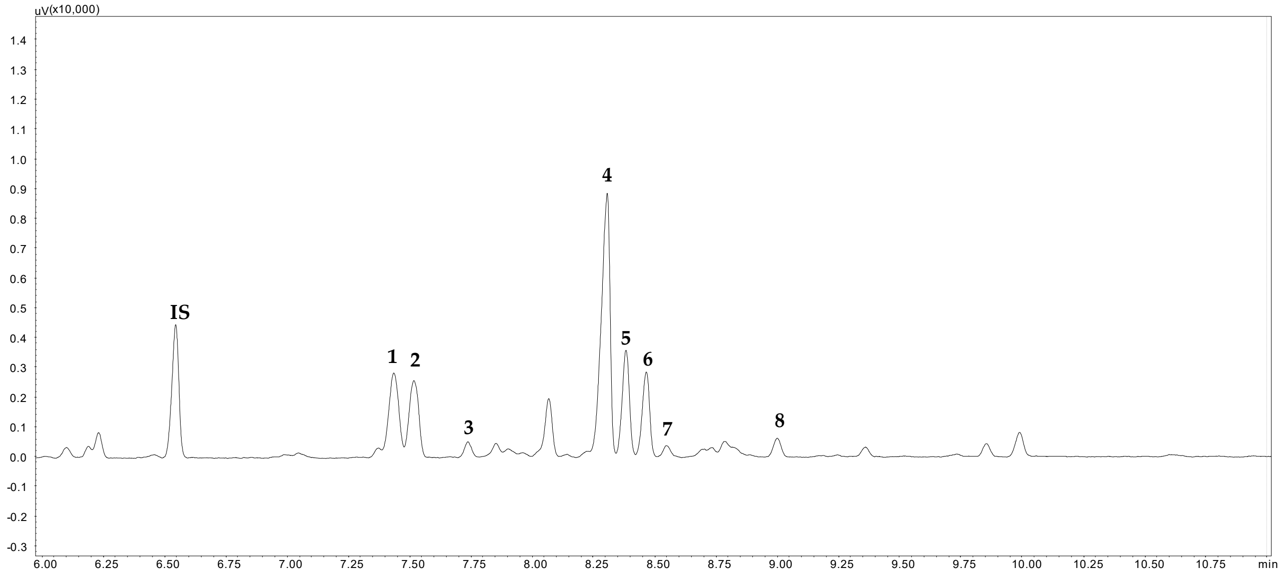

Supplement: Supplementary file 1 [file foods-12-03036-s001.zip › foods-2507570-supplementary.pdf]
